# Exploratory Graph Analysis for Factor Retention: Simulation Results for Continuous and Binary Data

**DOI:** 10.1177/00131644211059089

**Published:** 2021-12-28

**Authors:** Tim Cosemans, Yves Rosseel, Sarah Gelper

**Affiliations:** 1 Eindhoven University of Technology, The Netherlands; 2 Ghent University, Belgium

**Keywords:** exploratory factor analysis, factor retention, simulation, binary data, exploratory graph analysis

## Abstract

Exploratory graph analysis (EGA) is a commonly applied technique intended to help social scientists discover latent variables. Yet, the results can be influenced by the methodological decisions the researcher makes along the way. In this article, we focus on the choice regarding the number of factors to retain: We compare the performance of the recently developed EGA with various traditional factor retention criteria. We use both continuous and binary data, as evidence regarding the accuracy of such criteria in the latter case is scarce. Simulation results, based on scenarios resulting from varying sample size, communalities from major factors, interfactor correlations, skewness, and correlation measure, show that EGA outperforms the traditional factor retention criteria considered in most cases in terms of bias and accuracy. In addition, we show that factor retention decisions for binary data are preferably made using Pearson, instead of tetrachoric, correlations, which is contradictory to popular belief.

## Introduction

Social scientists often aim to explain behavioral phenomena using both observable and unobservable variables. To this aim, they frequently employ exploratory factor analysis (EFA), a technique that is used to discover and understand latent variables. With the goal of generating theory, researchers aim to explain a good portion of the variance among the originally measured J manifest variables by only using M factors (M<J) .

An important choice in the factor analytic process is the number of factors to retain. Extracting too few factors compresses variables into a smaller factor space leading to loss of information, neglect of important factors, distorted results, and increased error in the loadings. Extracting too many diffuses variables across a larger space. This results in splitting of factors, limiting interpretation, or trivial factors (Auerswald & Moshagen, 2019; Hayton et al., 2004).

Multiple studies have reviewed the use of EFA in psychological research (e.g., Conway & Huffcutt, 2003; Fabrigar et al., 1999; Ford et al., 1986; Goretzko et al., 2019; Henson & Roberts, 2006; Norris & Lecavalier, 2010). Almost all of these studies conclude that the choice regarding the number of factors to retain has been dominated by the standard options in statistical software. Goretzko et al. (2019), for example, find that 55% of studies still employ the Kaiser criterion and 46% the scree test. While it is important to choose a criterion that performs well in all circumstances, other methods, such as parallel analysis, or, more recently, exploratory graph analysis (EGA; Golino & Epskamp, 2017; Golino, Shi, et al., 2020) are often overlooked by applied researchers.

Research also increasingly relies on simpler response formats, such as questionnaires with binary responses, to increase response rates and decrease the danger of response bias. Instead of providing statements accompanied by a Likert-type scale, researchers simply ask the respondents whether or not they agree with the statement by either indicating “No” or “Yes”. For example, when asking respondents to position a brand with regard to certain characteristics, a question could be “Indicate to what extent you agree with the following statement: Brand X is innovative.” Dolnicar et al. (2011) show that a binary response format saves respondent time and is perceived simpler while not influencing reliability or interpretations of results. Yet, the validity of the results following from the application of traditional factor retention criteria to binary data has not been studied widely.

Several recent articles (see Table 1) have studied how the choice of factor retention criterion influences the correctness of the results. Most of these concentrate on continuous data (Auerswald & Moshagen, 2019; Crawford et al., 2010; Goretzko & Bühner, 2020; Li et al., 2020; Ruscio & Roche, 2012) or limit their focus to the assessment of parallel analysis and its variations (Cho et al., 2009; Green et al., 2016; Lim & Jahng, 2019). Only Yang and Xia (2015) assess various criteria in the context of ordinal and binary data, yet do not take into account the performance of EGA. The studies by Golino and Epskamp (2017) and Golino, Shi, et al. (2020) resemble our current investigation most closely, yet these authors do not determine the influence of the use of regular Pearson correlations for analyzing binary data, nor do they consider the performance of revised parallel analysis (Green et al., 2012). In addition, both studies simulate their data according to the factor model and do not allow for the influence of “minor factors” which model the “lack-of-fit” commonly found when fitting the factor model to real-world data (Hong, 1999; MacCallum & Tucker, 1991; Tucker et al., 1969).

**Table 1.** Overview of Recent Studies Reviewing Factor Retention Criteria.

Author	Dichotomous data	Correlations	Minor factors	EGA	Criteria included
Auerswald & Moshagen (2019)	No	Pearson	Yes	No	KC, PA, RPA, CD, $\chi^{2}$ test, Hull method, and empirical KC
Cho et al. (2009)	Yes	Pearson (dichotomous) and tetrachoric (dichotomous)	No	No	PA
Crawford et al. (2010)	No	Pearson	No	No	PA
Golino & Epskamp (2017)	Yes	Tetrachoric (dichotomous)	No	Yes	VSS, MAP, (E)BIC, PA, KC, and EGA
Golino, Christensen, & Moulder (2020)	Yes	Pearson (continuous) and tetrachoric (dichotomous)	No	Yes	KC, PA, scree plot, and EGA
Goretzko & Bühner (2020)	No	Pearson	No	No	KC, PA, CD, empirical KC, random forest, and extreme/automatic gradient boosting
Green et al. (2016)	Yes	Tetrachoric	No	No	PA and RPA
Li et al. (2020)	No	Pearson	No	No	PA, empirical KC, LRT, comparative fit index, Tucker–Lewis index, and RMSEA
Lim & Jahng (2019)	Yes	Pearson (continuous) and tetrachoric (dichotomous)	No	No	PA, RPA, and CD
Ruscio & Roche (2012)	No	Pearson	No	No	KC, PA, scree test, MAP, CD, AIC, BIC, and $\chi^{2}$ test
Yang & Xia (2015)	Yes	Tetrachoric	No	No	KC, PA, $\chi^{2}$ test, and fit indices
**This study**	**Yes**	**Pearson (continuous and dichotomous) and tetrachoric (dichotomous)**	**Yes**	**Yes**	**KC, scree plot, PA, RPA, MAP, and EGA**

*Note.* AIC = Akaike Information Criterion; EGA = exploratory graph analysis; KC = Kaiser criterion; PA = traditional parallel analysis; RPA = revised parallel analysis; CD = comparison data; VSS = very simple structure; MAP = minimum average partial; (E)BIC = (extended) Bayesian information criterion; LRT = likelihood ratio test; RMSEA = root mean square error of approximation.Matrices/vectors are in bold.

In what follows, we introduce the general factor analysis model, its assumptions and extensions for binary data, and discuss the various factor retention criteria considered more elaborately. Next, we illustrate the data simulation procedure and detail the fixed and variable input parameters. We then present the results of the study and discuss their impact. Limitations and opportunities for future research are discussed last.

## Factor Analysis

### Fundamental Equations

Underlying factor analysis is the assumption that the same latent variables influence the observed set of manifest variables, thereby causing a correlation structure between them (Bartholomew et al., 2008; Johnson & Wichern, 2002; Mulaik, 2009). Suppose we have J manifest variables that are centered around their mean as well as scaled by their standard deviation. The general linear factor model for M common factors can then be written as follows:

(1) $$Y_{j}=\sum_{m}\lambda_{\mathrm{jm}}\eta_{m}+\varepsilon_{j}\;\mathrm{for}\;j=1,2,\ldots ,J$$

Or, in matrix notation

(2) Y=ΛH+E

where Y is a J×1 vector of manifest variables, E is a J×1 vector representing the specific or unique factors, Λ is a J×M matrix with factor loadings, and H is a M×1 vector containing the common factors. From the covariances between the various $Y_{j}$ (which are equal to the correlations after centering and scaling), we can deduce the $\lambda_{\mathrm{jm}}$ , which are also the correlations between the latent and manifest variables if we scale the latent variables to have unit variance and the factors are constrained to be uncorrelated (Bartholomew et al., 2008; Johnson & Wichern, 2002; Mulaik, 2009; Russell, 2002).

Under certain assumptions, the fundamental theorem of factor analysis then states that the correlation matrix of the standardized observed variables $R_{\mathrm{YY}}$ can be written as follows:

(3) $$R_{\mathrm{YY}}=\Lambda R_{\mathrm{HH}}\Lambda \prime +\Psi$$

Given that the matrix Ψ is a diagonal one and contains the specific variances, the diagonal of $R_{\mathrm{YY}}-\Psi$ contains the communalities, which is the part of the variance explained by the common factors. The off-diagonal covariances are assumed to be solely due to the influence of the common factors (Johnson & Wichern, 2002; Mulaik, 2009).

### Factor Analysis for Binary Data

The model presented in Equation 1 is, however, not suited for binary variables. Pearson correlations are generally improper for studying dichotomous data: Given that we assume both $\varepsilon_{j}$ and $\eta_{m}$ to be continuous, their combination, $Y_{j}$ can take on any value and is not constrained to the set {0,1} . To address this limitation, we can apply the underlying variable approach. This assumes that the observed binary variable $Y_{j}$ is only a partial realization of the continuous variable $Y_{j}^{*}{=}^{d}N(0,1)$ that indicates whether $Y_{j}^{*}$ exceeds a certain threshold $\delta_{j}$ . This assumption allows us to treat the $Y_{j}^{*}$ as if they were generated by the factor model from Equation 1 (Bartholomew et al., 2008; Holgado-Tello et al., 2010; Olsson, 1979). Implementing this procedure requires estimating the matrix of tetrachoric correlations (Olsson, 1979) to be used in the factor analytic process.

### Factor Retention Criteria

Previous research (e.g., Conway & Huffcutt, 2003; Fabrigar et al., 1999; Ford et al., 1986; Goretzko et al., 2019; Henson & Roberts, 2006; Norris & Lecavalier, 2010) has shown that the most frequently employed methods are the eigenvalue-greater-than-one rule or Kaiser criterion (Kaiser, 1960), scree plot (Cattell, 1966), parallel analysis (Horn, 1965), and minimum average partial method (Velicer, 1976). In this section, we will introduce all of these methods, along with their extensions. In addition, we will also look at the recently developed EGA (Golino & Epskamp, 2017; Golino, Shi, et al., 2020).

#### Kaiser Criterion

The majority of factor extraction criteria rely on the eigenvalues of the observed correlation matrix. Starting from the factor model in Equation 1 and the corresponding decomposition of the correlation matrix $R_{\mathrm{YY}}$ in Equation 3, the common factor model estimates Λ such that

(4) $$R_{\mathrm{YY}-C}=\Lambda \Lambda^{\prime }$$

where $R_{\mathrm{YY}-C}=R_{\mathrm{YY}}-\Psi$ is the reduced correlation matrix with communalities as diagonal elements (i.e., 1—unique variance). This can be done by decomposing the reduced correlation matrix into its eigenvalues and eigenvectors. If we only extract m factors, where m is smaller than the rank of $R_{\mathrm{YY}-C}$ , we only use the first m eigenvalues and eigenvectors. This entails the above equation will only hold approximately instead of exactly (Johnson & Wichern, 2002). The principal components model, which is most often employed, estimates Λ in a similar way. It, however, replaces the reduced correlation matrix $R_{\mathrm{YY}-C}$ with the full correlation matrix $R_{\mathrm{YY}}$ . As a consequence, each of the eigenvalues can be interpreted as the variance explained by the corresponding component (Auerswald & Moshagen, 2019). Kaiser (1960) states that extracting additional factors is warranted as long as their eigenvalues are greater than one, arguing that a factor should at least explain as much variance as a single item.

#### Scree Plot

Cattell’s scree test (Cattell, 1966) involves plotting the sequential eigenvalues from the factor analysis procedure and looking for the “elbow” in the graph. This point defines the optimal number of factors to retain.

The subjectivity involved in this criterion is, however, high as it is merely based on a visual interpretation (Hayton et al., 2004). Raîche et al. (2013) therefore devise several nongraphical solutions to this problem, among which the acceleration factor, which closely aligns with the intuition behind the scree test. More specifically, Raîche et al. (2013) state that the elbow of the plot corresponds to the point where the slope of the curve changes most abruptly. The number of factors to retain is the point preceding the number of factors where the acceleration factor is maximal.

This solution does, however, not take into account that the eigenvalues of the retained factors could be so small as to render them useless. In addition, the criterion is therefore often evaluated in combination with the Kaiser criterion discussed above. This amounts to taking the minimum of the factors indicated by either one of these rules (Raîche et al., 2013). Again the eigenvalues used for these tests come from the components analysis of the correlation matrix.

#### Traditional Parallel Analysis

Because of sampling error, eigenvalues can be larger than one even if no additional factor is present. This causes the Kaiser criterion to regularly overextract (Auerswald & Moshagen, 2019).

Parallel analysis, originally presented by Horn (1965), illustrates the concept of using the eigenvalues of random data with no underlying factor structure to determine the optimal number of factors to retain. The procedure starts by simulating k data sets (100 in this study) comprising completely random numbers from a standard normal distribution with the same number of variables and observations as the user-provided data set. It then calculates the eigenvalues of the correlation matrix for each of these k data sets. For each mth eigenvalue the procedure now generated a distribution of eigenvalues corresponding to the null hypothesis of a nonexistent mth factor. The eigenvalues of the original data set can then be compared with either the mean eigenvalue for each factor m or its 95th percentile (in this study we will compare it with both). As long as the empirical eigenvalue is above its simulated counterpart, the inclusion of an additional factor is warranted (Hayton et al., 2004; O’Connor, 2000).

Given that parallel analyisis using principal components analysis has often been found to outperform its common factor counterpart, we will be using the former method (Auerswald & Moshagen, 2019; Crawford et al., 2010; Golino, Shi, et al., 2020; Ruscio & Roche, 2012).

#### Revised Parallel Analysis

Green et al. (2012) argue that the original approach to parallel analysis is more of a heuristic than a mathematically rigorous procedure. They reason that the method described above only yields the appropriate reference distribution for the first empirical eigenvalue: The eigenvalue for deciding upon the m+1th factor needs to be compared with the distribution of the m+1th eigenvalue from a sample with m underlying factors and not zero, as is the case in the traditional method. Revised parallel analysis therefore starts with the appropriate reference distribution by sampling from a population with only m underlying factors.

As recommended by Green et al. (2012), we apply the revised parallel analysis both with and without the addition of the Kaiser criterion and based on the 95th percentile of eigenvalues. In addition, recent simulations by Auerswald and Moshagen (2019) have shown that if based on the common factor model, the method severely underperforms. Therefore, implementation in this study is based on principal components analysis.

#### Minimum Average Partial

Velicer (1976) proposes choosing the optimal number of factors based on the minimum average squared partial correlation. Given J manifest variables, and a partial correlation matrix A with elements $a_{\mathrm{vw}}$ , for each number of factors m , the following summary statistic is computed

(5) $$f_{m}=\sum \sum_{v\neq w}\left[\frac{{\left(a_{\mathrm{vw}}^{*}\right)}^{2}}{J(J-1)}\right]$$

which is the average of the squared partial correlations (only including the off-diagonal elements) after the first m components are partialed out. The optimal number of factors is defined as the point where $f_{m}$ reaches a minimum, unless $f_{1}>f_{0}$ , which is defined as follows:

(6) $$f_{0}=\sum \sum_{v\neq w}\left[\frac{{\left(a_{\mathrm{vw}}\right)}^{2}}{J(J-1)}\right]$$

in which case no components are extracted (Velicer, 1976). This means that factors are retained as long as the variance left in the partial (co)variance matrix is systematic in nature (Hayton et al., 2004).

#### EGA

Unlike most methods discussed above, EGA, developed by Golino and Epskamp (2017), does not rely on the eigenvalues of the (reduced) correlation matrix. It, therefore, is not impacted by a preliminary choice of model (common components or factor analysis) or method (principal components, principal axis factor, maximum likelihood, etc.) and any problems this might entail (such as violations of assumptions). Instead, it models the variables as a multivariate normally distributed network (i.e., a Gaussian graphical model or GGM; Golino & Epskamp, 2017). The algorithm represents the manifest variables as a network of nodes connected by weighted edges and proposes the latent variables from the factor model will cause these nodes to cluster together. Golino and Epskamp (2017) use the inverse variance–covariance matrix between manifest variables to represent the edge weights, which, after standardization, can be interpreted as a matrix of partial correlation coefficients. While the variance–covariance matrix can be inverted directly, doing so entails larger standard errors and unstable parameters in small data sets due to overfitting. Therefore, Golino and Epskamp (2017) propose the use of the LASSO to estimate this matrix. As this type of penalized maximum likelihood estimates many coefficients to be exactly zero, it guards against overfitting. The tuning parameter of this method, that controls the degree of sparsity, is estimated by minimizing the extended Bayesian information criterion (Golino & Epskamp, 2017; Golino, Shi, et al., 2020).

The Walktrap algorithm then allows detecting of the number of dimensions in the network. If we let A be a square matrix of edge weights (i.e., partial correlations between manifest variables), we can denote $a_{\mathrm{vw}}$ as the strength of the partial correlation between nodes v and w . The strength of node v can then be denoted as $NS_{v}=\sum_{w}a_{\mathrm{vw}}$ and the transition matrix containing the uniform probabilities of moving from one node to another as the matrix P with elements $p_{\mathrm{vw}}=a_{\mathrm{vw}}/NS_{v}$ . This matrix can be used to compute the distance between nodes as follows:

(7) $$d_{\mathrm{vw}}=\sqrt{\sum_{k=1}^{n}\frac{{\left(p_{\mathrm{vk}}-p_{\mathrm{wk}}\right)}^{2}}{NS_{k}}}$$

with n representing the total number of nodes involved. If we define the transition probabilities between a node or manifest variable and a community (C) or factor as

(8) $$p_{\mathrm{Cw}}=\frac{1}{\left|C\right|}\sum_{v\in C}p_{\mathrm{vw}}$$

we can be further expand the previous equation to include the distance between two communities as

(9) $$d_{C_{1}C_{2}}=\sqrt{\sum_{k=1}^{n}\frac{{\left(p_{C_{1}k}-p_{C_{2}k}\right)}^{2}}{NS_{k}}}$$

The algorithm starts with each node as a cluster, calculating the distances between them and joining two clusters at a time, each time recalculating the distances between the nodes and the clusters. The choice of which clusters to merge depends on the change in variation (σ) that would be induced by merging two clusters $C_{1}$ and $C_{2}$ into a new cluster $C_{3}$ , calculated as

(10) $$\Delta \sigma (C_{1},C_{2})=\frac{1}{n}\left(\sum_{v\in C_{3}}r_{vC_{3}}^{2}-\sum_{v\in C_{1}}r_{vC_{1}}^{2}-\sum_{v\in C_{2}}r_{vC_{2}}^{2}\right)$$

This variation is minimized in each step.

The best number of clusters is chosen based on the number that maximizes modularity. If we have a network with two clusters j and k and define the fraction of the edges within a cluster j as $e_{\mathrm{jj}}$ so that $\sum_{j}e_{\mathrm{jj}}=1$ , then a division of a network into clusters is meaningful if $e_{\mathrm{jj}}+e_{\mathrm{kk}}$ is maximized. Yet, this index is not informative when there is only one cluster, in which case the maximal value is 1 (also the value of $\sum_{j}e_{\mathrm{jj}}$ ). This can be circumvented by subtracting the value the index would take if the edges were placed a random, so that we can define the modularity for a given cluster j as

(11) $$Q_{j}=\sum_{j}\left[e_{\mathrm{jj}}-{\left(\sum_{i}e_{\mathrm{ij}}\right)}^{2}\right]$$

This criterion penalizes network structures with only one cluster. As a consequence, this algorithm is not expected to work well for unidimensional structures. Golino, Shi, et al. (2020) therefore start their improved version of the algorithm by simulating a unidimensional data set with four variables and loadings of 0.70 and bind these simulated data with the user-provided data. This prevents the penalization inherent to the Walktrap algorithm for single cluster solutions (Golino, Shi, et al., 2020). Implementation of this algorithm is done by means of the *EGAnet* package in *R* (Golino, Christensen, & Moulder, 2020).

## Simulation Setup

### Population and Data Generating Process

In this section, we describe the procedure that generates population correlation matrices from which the data for factor analysis are sampled. This procedure relies on the simulation model, proposed by Tucker et al. (1969), that shares many features with the formal model of factor analysis discussed previously. Both models assume the existence of a major domain that contains $M_{1}$ factors of influence the researcher wishes to study. However, the formal model only assumes the additional impact of unique factors that account for variable-specific influences and measurement errors. The simulation model proposes the further influence of (infinitely) many $\left(M_{2}\right)$ minor factors not modeled by the researcher. As is often the case, the researcher only aims to explain the major influences at play using the most parsimonious model. She often ignores those variables that only explain a minor part of the observed variance and could be costly to measure. In this scenario, the common factor model will not exactly fit the data at hand (Hong, 1999; MacCallum & Tucker, 1991).

In this study, we require the major factor loadings to have a perfect simple structure, following the applications of this method in literature (Briggs & MacCallum, 2003; De Winter & Dodou, 2012, 2016; MacCallum et al., 1999; Pearson & Mundform, 2010). This is a factor solution for which each factor only has a subset of variables with high loadings and each variable has high loadings on only some factors, but preferably only one (Fabrigar et al., 1999).

A matrix with loadings on the several other, unmodelled, minor factors is generated by drawing independent random standard normal deviates ( μ=0 , σ=1 ). These entries are multiplied columnwise by a constant $(1-\epsilon {)}^{\left(m_{2}-1\right)}$ to form a decreasing geometric series. In this way, the infinite number of minor factors can be approximated by a finite matrix. In addition, the rows of the resulting matrix are normalized to have unit length (Hong, 1999; MacCallum & Tucker, 1991; Tucker et al., 1969). Further details on how the population correlation matrix was generated are described in the Supplementary Materials.

Given this correlation matrix $R_{\mathrm{YY}}$ , we can use the procedure by Kaiser and Dickman (1962) to simulate data with sample size N by

(12) Z=XF′

where X is an N×J matrix with independent randomly drawn standard normal elements ( μ=0 , σ=1 ) and $F=UD^{1/2}$ , that is, the multiplication of the eigenvectors of $R_{\mathrm{YY}}$ (denoted by U ) and the square root of the diagonal matrix containing the eigenvalues of $R_{\mathrm{YY}}$ (denoted by D ). The resulting matrix Z then contains observations from a multivariate normal distribution with mean zero and unit standard deviations, the sample correlation matrix of which can be generated in the usual way (Hong, 1999). These items are subsequently dichotomized by substituting all values lower than a prespecified cut-off value δ by a zero and all values higher by a one, which is common practice in simulation research using dichotomous data (e.g., Barendse et al., 2015).

### Scenarios

Several parameters will be varied throughout the study, their combinations constitute the different scenarios that will be analyzed. More specifically, sample size (N=100;1,000) , interfactor correlations between major factors (Θ=0;0.5) , and communalities from major factors (in the range 0.2−0.3−0.4 or 0.6−0.7−0.8 ) will be altered, resulting in a 2×2×2 design (Table 2). This design results in four different population correlation matrices (defined by the interfactor correlations and communalities from major factors). Both these and the accompanying factor loading matrices can be found in the Supplementary Materials. For each of the scenarios defined by the combination of these three variables 1,000 samples are generated, resulting in a total of 2×2×2×1,000=8,000 samples. Each of which will be analyzed in three ways:

**Table 2.** Variable Input Parameters.

Parameter	Levels	References
Sample size (N)	100/1,000	Henson & Roberts (2006)
Interfactor correlations (Θ)	0/0.5	De Winter & Dodou (2016)
Communalities from major factors $\left(b_{1j}^{2}\right)$ ^a^	0.2−0.3−0.4/0.6−0.7−0.8	Tucker et al. (1969)

a Communality levels from major factors considered are two ranges. Actual communalities used for each of the variables are randomly sampled from this range (following Tucker et al. (1969)).

- Directly as continuous data using a Pearson correlation matrix;
- After dichotomization resulting in a 50–50 split between zeroes and ones (δ=0) , using both the Pearson and tetrachoric correlation matrices;
- After dichotomization resulting in a 75–25 split between zeroes and ones (δ≈0.67) , using both the Pearson and tetrachoric correlation matrices.

**Figure 1.**
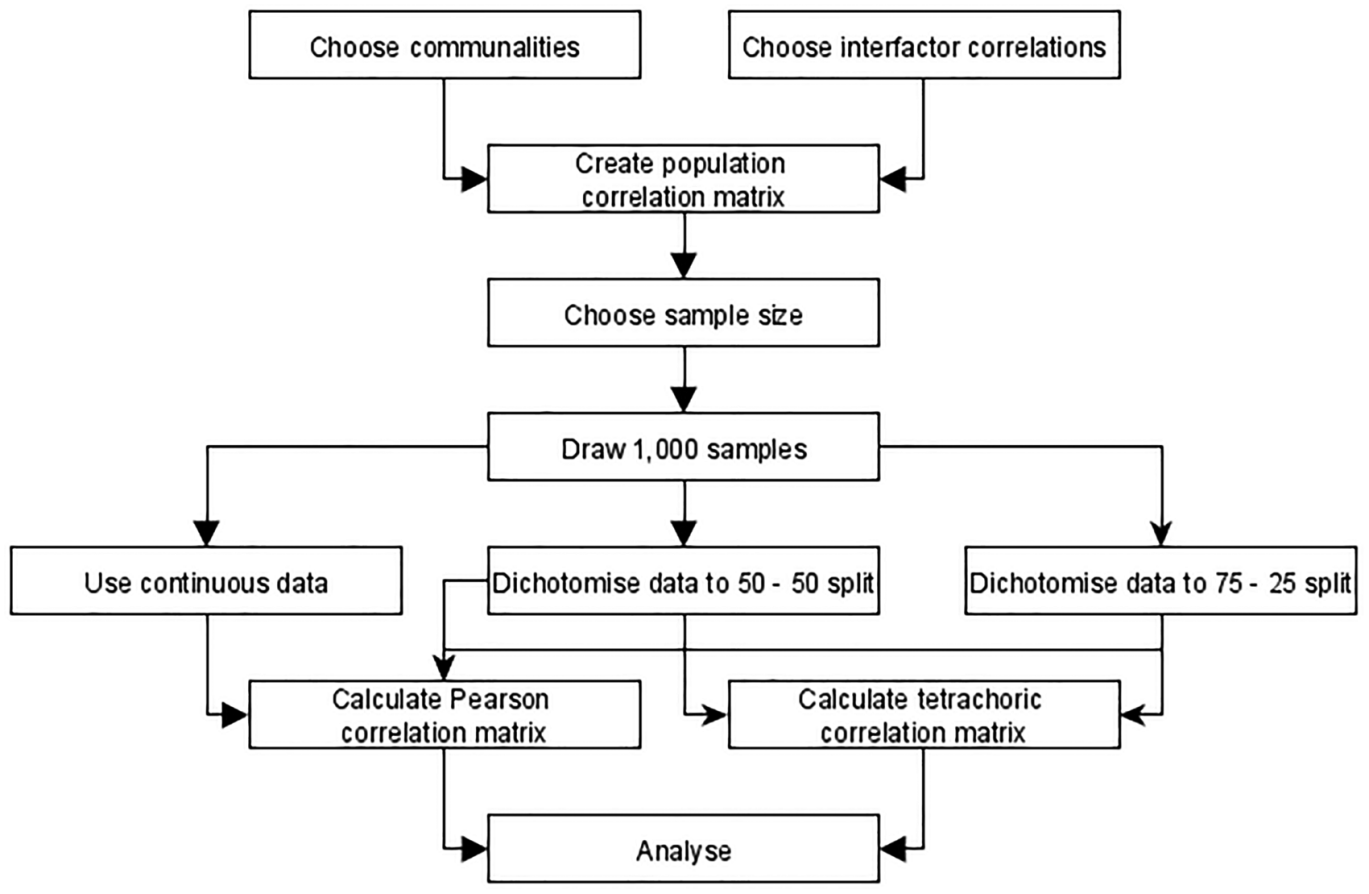
Schematic Representation of the Data Simulation Procedure.

The comparison of the results between the continuous and dichotomous analyses allows us to assess the impact of the dichotomization on the results of the factor analytic process. Academic research usually sets the cut-off for this dichotomization at δ=0 , resulting in a 50–50 split between zeroes and ones (assuming normally distributed data; e.g., Barendse et al., 2015). In practice, the split is often more skewed. We therefore also investigate a 75–25 division, leading to δ≈0.67 . In addition, binary data are still often analyzed using regular Pearson correlations instead of its tetrachoric counterpart (Holgado-Tello et al., 2010), to assess the impact of this decision, we analyze the binary samples in both ways. A schematic summary of this workflow is represented in Figure 1.

In addition, some parameters will remain fixed throughout the study. Their values will not be changed as to assess their impact (Table 3). We keep the number of variables (J) fixed at 20, and the number of major factors $\left(M_{1}\right)$ at 3. Following De Winter and Dodou (2016), we set the number of minor factors $\left(M_{2}\right)$ to 200. The common ratio ( ϵ ) is set to 0.8 (Hong, 1999); the correlation between both the minor factors and the major and minor factors is set to 0 (Mulaik, 2009).

**Table 3.** Fixed Input Parameters.

Parameter	Value	References
Variables (J)	20	Goretzko et al. (2019)
Major factors $\left(M_{1}\right)$	3	Goretzko et al. (2019)
Minor factors $\left(M_{2}\right)$	200	De Winter & Dodou (2016)
Unique factors $\left(M_{3}\right)$	20 (= J )	Tucker et al. (1969)
Common ratio ( 1−ϵ )	0.8	Hong (1999)
Correlation minor factors^a^	I	Mulaik (2009)
Correlation major and minor factors^a^	0	Mulaik (2009)

a The factor analytic model assumes all unmodelled influences to be uncorrelated with those that are (Mulaik, 2009).

The communalities from the minor factors are always set to half of the square root of the variance not explained by the communalities from the major factors, following Tucker et al. (1969). The rest of the variance is taken to be explained by the unique variances. For more info on the data generation process, please refer to the Supplementary Materials.

### Analysis

Data and accompanying correlation matrices are generated as described in Figure 1 and analyzed using nine different factor retention criteria. Every population correlation matrix represents a hypothetical population factor model characterized by certain conditions that applied researchers want to unravel. Yet, researchers only observe one sample from this population at a time. We draw 2,000 samples from each scenario ( 1,000 with N=100 and 1,000 with N=1,000 ) and decide on the dichotomization and skewness of the data. Each of the resulting data sets are subsequently analyzed as if they resulted from empirical research: We decide on the type of correlation matrix to use, the factor retention criterion, and record the recommended number of factors to retain. Afterward, two new variables are created: a dummy, indicating whether the criterion returned the correct number of factors (three in this case), and the bias, which corresponds to the predicted number of factors minus the real number. After running the simulation, both variables are used as dependent variables in a logistic and linear regression, respectively, with dummies for the sample size, interfactor correlations, communalities from major factors, type of data, type of correlations used, and factor retention criterion as independent variables as well as interactions between the criteria and the remainder of the variables. This results in a fully saturated regression model which can be used to predict the expected bias and probability of correctly predicting the number of factors in each of the scenarios. Full regression results are displayed in the Supplementary Materials. In the following paragraphs, we will discuss these results graphically to assess the best criterion under various circumstances.

Hereafter, each of the criteria will be abbreviated in the following way: EV: Kaiser criterion, AF: acceleration factor alternative to the scree plot, AFEV: acceleration factor combined with the Kaiser criterion, PAM: parallel analysis based on the mean eigenvalues, PA95: parallel analysis based on the 95th percentile of eigenvalues, RPA: revised parallel analysis based on the 95th percentile of eigenvalues, RPAEV: revised parallel analysis based on the 95th percentile of eigenvalues combined with the Kaiser criterion, MAP: minimum average partial method, and EGA: exploratory graph analysis.

## Results

### Continuous Data

**Figure 2.**
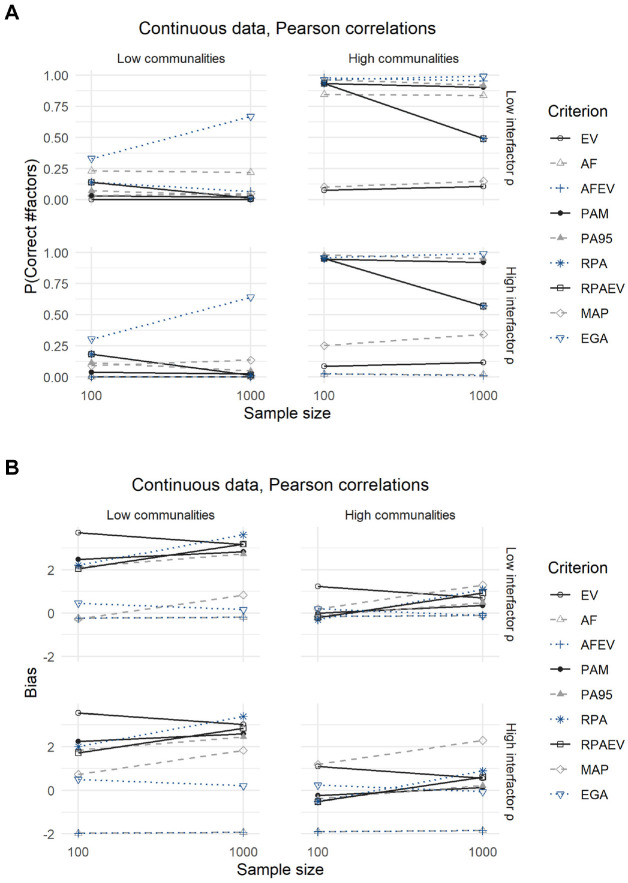
Results for Continuous Data: (A) Expected Probability of Correctly Predicting the Number of Factors and (B) Expected Bias. *Note.* EV = Kaiser criterion; AF = acceleration factor alternative to the scree plot; AFEV = acceleration factor combined with the Kaiser criterion; PAM = parallel analysis based on the mean eigenvalues; PA95 = parallel analysis based on the 95th percentile of eigenvalues; RPA = revised parallel analysis based on the 95th percentile of eigenvalues; RPAEV = revised parallel analysis based on the 95th percentile of eigenvalues combined with the Kaiser criterion; MAP = minimum average partial method; EGA = exploratory graph analysis.

The linear and logistic regression models can be used to predict the expected bias and probability of correctly predicting the number of factors for each of the nine criteria. The results of this procedure for continuous data are displayed in Figure 2 and Table 4.

**Table 4.** Results for Continuous Data.

A. Expected probability of correctly predicting the number of factors											
Communalities	Interfactor correlation	*N*	EV	AF	AFEV	PAM	PA95	RPA	RPAEV	MAP	EGA
Low	Low	100	0	0.23	0.14	0.03	0.07	0.14	0.14	0.03	0.33
Low	Low	1,000	0	0.22	0.07	0.02	0.03	0.01	0.01	0.05	0.67
Low	High	100	0	0	0	0.04	0.11	0.18	0.18	0.09	0.3
Low	High	1,000	0	0	0	0.03	0.05	0.02	0.02	0.13	0.64
High	Low	100	0.08	0.85	0.98	0.93	0.97	0.93	0.93	0.1	0.96
High	Low	1,000	0.11	0.84	0.95	0.9	0.92	0.49	0.49	0.15	0.99
High	High	100	0.09	0.02	0.03	0.95	0.98	0.95	0.95	0.25	0.96
High	High	1,000	0.12	0.02	0.01	0.92	0.95	0.57	0.57	0.34	0.99
B. Expected bias											
Communalities	Interfactor correlation	*N*	EV	AF	AFEV	PAM	PA95	RPA	RPAEV	MAP	EGA
Low	Low	100	3.69	−0.23	−0.24	2.47	2.13	2.21	2.05	−0.26	0.46
Low	Low	1,000	3.16	−0.19	−0.19	2.83	2.72	3.6	3.17	0.84	0.16
Low	High	100	3.55	−1.97	−1.98	2.23	1.85	2	1.72	0.73	0.49
Low	High	1,000	3.02	−1.93	−1.93	2.6	2.45	3.39	2.85	1.83	0.2
High	Low	100	1.23	−0.15	−0.16	0	−0.11	−0.3	−0.2	0.2	0.19
High	Low	1,000	0.7	−0.11	−0.11	0.36	0.49	1.09	0.93	1.3	−0.11
High	High	100	1.09	−1.89	−1.9	−0.24	−0.38	−0.51	−0.52	1.19	0.23
High	High	1,000	0.56	−1.85	−1.86	0.12	0.21	0.88	0.61	2.29	−0.07

*Note.* EV = Kaiser criterion; AF = acceleration factor alternative to the scree plot; AFEV = acceleration factor combined with the Kaiser criterion; PAM = parallel analysis based on the mean eigenvalues; PA95 = parallel analysis based on the 95th percentile of eigenvalues; RPA = revised parallel analysis based on the 95th percentile of eigenvalues; RPAEV = revised parallel analysis based on the 95th percentile of eigenvalues combined with the Kaiser criterion; MAP = minimum average partial method; EGA = exploratory graph analysis.

The well-known EV criterion is among the worst performing in each of the scenarios as it never correctly predicts the number of factors to retain in case communalities from major factors are low. The expected probability of a correct prediction rises only slightly, to approximately 0.10, when communalities from major factors or sample sizes increase. The same can be said for the MAP, AF, and AFEV criteria, with the exception that the latter two perform seemingly well in scenarios defined by high communalities from major factors and low interfactor correlations where they reach expected probabilities of a correct prediction of 0.84 to 0.98. At the other end of the spectrum is the EGA criterion, which has the highest expected hit rates of all criteria in each of the scenarios. Even in challenging circumstances of low communalities from major factors where most other criteria fail, does the EGA method achieve a reasonable expected accuracy of around 0.65 with larger sample sizes. Results regarding other criteria are less clear-cut as their performance depends on the scenarios in which they are employed. Both PAM and PA95 perform well in situations with high communalities from major factors, approximating the same expected accuracy as the EGA criterion in the range 0.90 to 0.98. When variables are only weakly influenced by their common factors, both methods underperform and have expected accuracies similar to that of the EV criterion in the range 0.02 to 0.11. Finally, both RPA and RPAEV show patterns contrasting those of other criteria, with expected accuracies decreasing as sample sizes increase. When faced with small samples, RPA and RPAEV approach the performance of the EGA, especially when communalities from major factors are high. In larger samples their accuracy decreases significantly to 0.57 in the best case scenario of high communalities from major factors and high interfactor correlations.

The second panel of Figure 2 and Table 4 indicates to which extent the underperforming criteria favor over- or underextraction (indicated by positive and negative expected biases, respectively). The EV criterion almost always overextracts, a result also discussed Hayton et al. (2004), and at its worst predicts almost four factors more than optimal. This is in contrast to the AF and AFEV criteria, which tend to favor less factors when they underperform as their expected biases are all negative. The PAM, PA95, RPA, RPAEV, and MAP commonly overestimate the optimal number of factors when they do indicate the incorrectness of the number of factors. Again, the expected bias of the EGA criterion is small in all scenarios.

### Dichotomous Data (50–50 Split)

**Figure 3.**
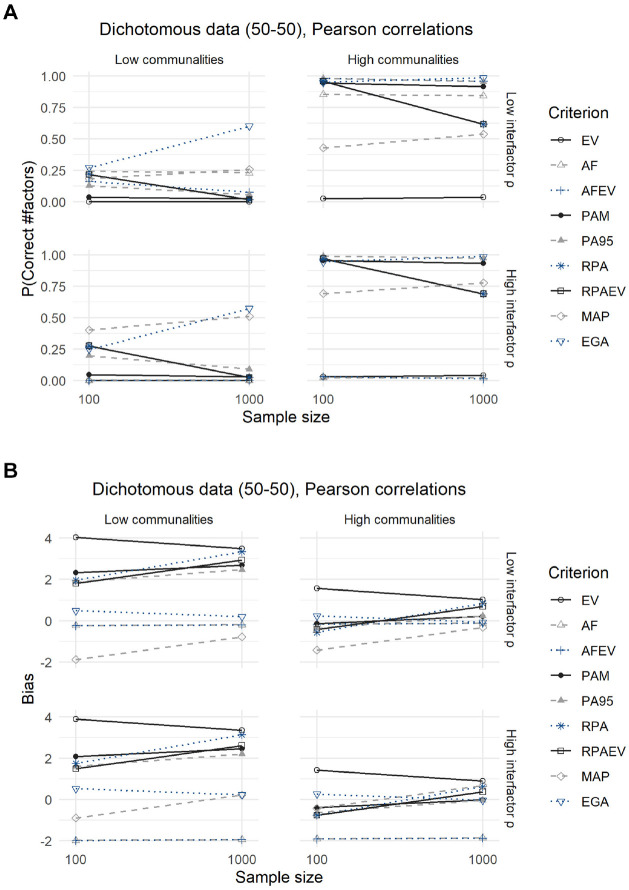
Results for Dichotomous Data With a 50–50 Split, Using Pearson Correlations: (A) Expected Probability of Correctly Predicting the Number of Factors and (B) Expected Bias.

**Figure 4.**
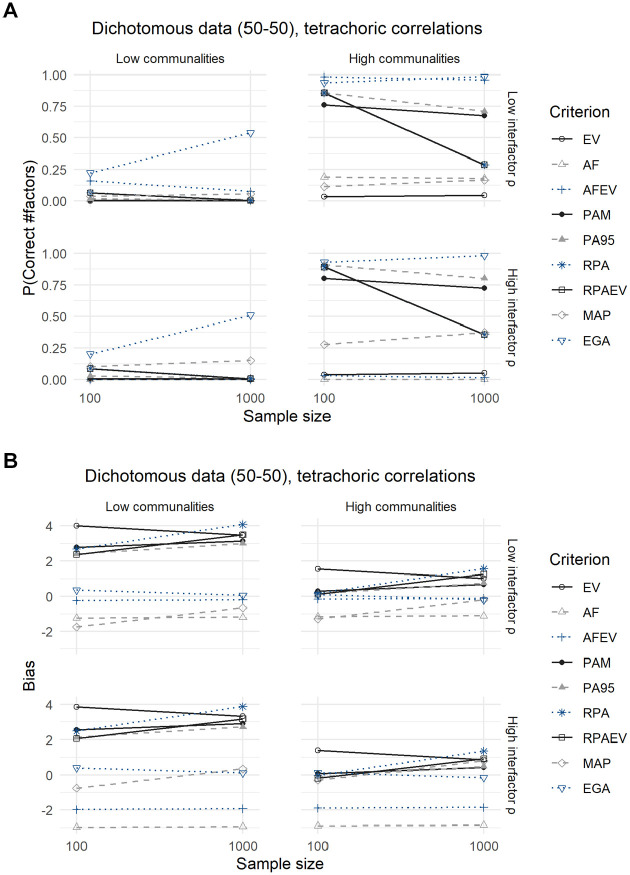
Results for Dichotomous Data With a 50–50 Split, Using Tetrachoric Correlations: (A) Expected Probability of Correctly Predicting the Number of Factors and (B) Expected Bias.

Both Figures 3 and 4 and their corresponding Tables 5 and 6 show the results for dichotomous data with a 50–50 split between zeroes and ones. The difference being that the latter displays the outcomes for the analysis using tetrachoric correlations, and the former using Pearson correlations.

**Table 5.** Results for Continuous Data With a 50–50 Split, Using Pearson Correlations.

A. Expected probability of correctly predicting the number of factors											
Communalities	Interfactor correlation	*N*	EV	AF	AFEV	PAM	PA95	RPA	RPAEV	MAP	EGA
Low	Low	100	0	0.24	0.16	0.04	0.13	0.22	0.22	0.18	0.27
Low	Low	1,000	0	0.23	0.08	0.02	0.06	0.02	0.02	0.26	0.6
Low	High	100	0	0	0	0.05	0.2	0.28	0.28	0.4	0.25
Low	High	1,000	0	0	0	0.03	0.09	0.03	0.03	0.51	0.57
High	Low	100	0.03	0.85	0.98	0.95	0.98	0.96	0.96	0.43	0.95
High	Low	1,000	0.04	0.84	0.96	0.92	0.96	0.62	0.62	0.54	0.99
High	High	100	0.03	0.02	0.03	0.96	0.99	0.97	0.97	0.69	0.94
High	High	1,000	0.04	0.02	0.01	0.93	0.97	0.69	0.69	0.78	0.99
B. Expected bias											
Communalities	Interfactor correlation	*N*	EV	AF	AFEV	PAM	PA95	RPA	RPAEV	MAP	EGA
Low	Low	100	4.03	−0.24	−0.25	2.33	1.88	1.95	1.81	−1.88	0.49
Low	Low	1,000	3.49	−0.2	−0.21	2.69	2.47	3.34	2.94	−0.78	0.19
Low	High	100	3.89	−1.98	−1.99	2.09	1.6	1.74	1.49	−0.89	0.53
Low	High	1,000	3.36	−1.94	−1.95	2.45	2.2	3.14	2.61	0.21	0.23
High	Low	100	1.57	−0.16	−0.17	−0.15	−0.36	−0.56	−0.43	−1.42	0.22
High	Low	1,000	1.03	−0.12	−0.13	0.21	0.24	0.83	0.7	−0.32	−0.07
High	High	100	1.43	−1.9	−1.91	−0.38	−0.63	−0.76	−0.75	−0.43	0.26
High	High	1,000	0.9	−1.86	−1.87	−0.02	−0.03	0.63	0.37	0.67	−0.03

**Table 6.** Results for Dichotomous Data With a 50–50 Split, Using Tetrachoric Correlations.

A. Expected probability of correctly predicting the number of factors											
Communalities	Interfactor correlation	*N*	EV	AF	AFEV	PAM	PA95	RPA	RPAEV	MAP	EGA
Low	Low	100	0	0.01	0.16	0.01	0.02	0.06	0.06	0.04	0.22
Low	Low	1,000	0	0.01	0.08	0	0.01	0	0	0.06	0.54
Low	High	100	0	0	0	0.01	0.03	0.09	0.09	0.1	0.2
Low	High	1,000	0	0	0	0.01	0.01	0.01	0.01	0.15	0.51
High	Low	100	0.03	0.19	0.98	0.76	0.86	0.86	0.86	0.11	0.94
High	Low	1,000	0.05	0.18	0.96	0.68	0.71	0.29	0.29	0.17	0.98
High	High	100	0.04	0	0.03	0.8	0.91	0.89	0.89	0.28	0.93
High	High	1,000	0.05	0	0.01	0.72	0.8	0.36	0.36	0.37	0.98
B. Expected bias											
Communalities	Interfactor correlation	*N*	EV	AF	AFEV	PAM	PA95	RPA	RPAEV	MAP	EGA
Low	Low	100	4	−1.24	−0.23	2.78	2.4	2.68	2.37	−1.75	0.36
Low	Low	1,000	3.47	−1.2	−0.19	3.14	2.99	4.08	3.5	−0.65	0.06
Low	High	100	3.86	−2.98	−1.97	2.55	2.12	2.48	2.05	−0.76	0.4
Low	High	1,000	3.33	−2.94	−1.93	2.91	2.72	3.87	3.17	0.34	0.1
High	Low	100	1.54	−1.16	−0.15	0.31	0.16	0.18	0.13	−1.3	0.09
High	Low	1,000	1.01	−1.12	−0.11	0.67	0.76	1.57	1.25	−0.2	−0.2
High	High	100	1.4	−2.9	−1.9	0.07	−0.11	−0.03	−0.2	−0.3	0.13
High	High	1,000	0.87	−2.86	−1.85	0.43	0.49	1.36	0.93	0.8	−0.16

*Note.* EV = Kaiser criterion; AF = acceleration factor alternative to the scree plot; AFEV = acceleration factor combined with the Kaiser criterion; PAM = parallel analysis based on the mean eigenvalues; PA95 = parallel analysis based on the 95th percentile of eigenvalues; RPA = revised parallel analysis based on the 95th percentile of eigenvalues; RPAEV = revised parallel analysis based on the 95th percentile of eigenvalues combined with the Kaiser criterion; MAP = minimum average partial method; EGA = exploratory graph analysis.

Using Pearson correlations, the expected probabilities of accurately predicting the number of factors are nearly identical to those obtained from the analysis of continuous data. One exception is the MAP criterion, which now performs significantly better in almost every scenario and even outperforms the EGA method when faced with small samples, low communalities from major factors and high interfactor correlations. In this case its expected accuracy rises to 0.4 compared with 0.25 for EGA. Looking at the second panel of Figure 3, the differences are again minimal. The expected biases of the EV criterion rise and those of PA95 and RPA decrease slightly. Only the MAP criterion becomes significantly less biased.

Results change when we analyze the data using tetrachoric correlations (Figure 4 and Table 6). More specifically, the expected accuracies of all criteria shift downward but the relative performances remain similar to the continuous case. Remarkably, the MAP criterion loses its edge over the other criteria when using tetrachoric correlations. In terms of expected bias, conclusions are very similar to those drawn earlier when analyzing continuous data. However, the expected bias of the MAP criterion has shifted further downward in comparison the case of binary data and an analysis with Pearson correlations. It, however, still outperforms the case of continuous data.

### Dichotomous Data (75–25 Split)

**Figure 5.**
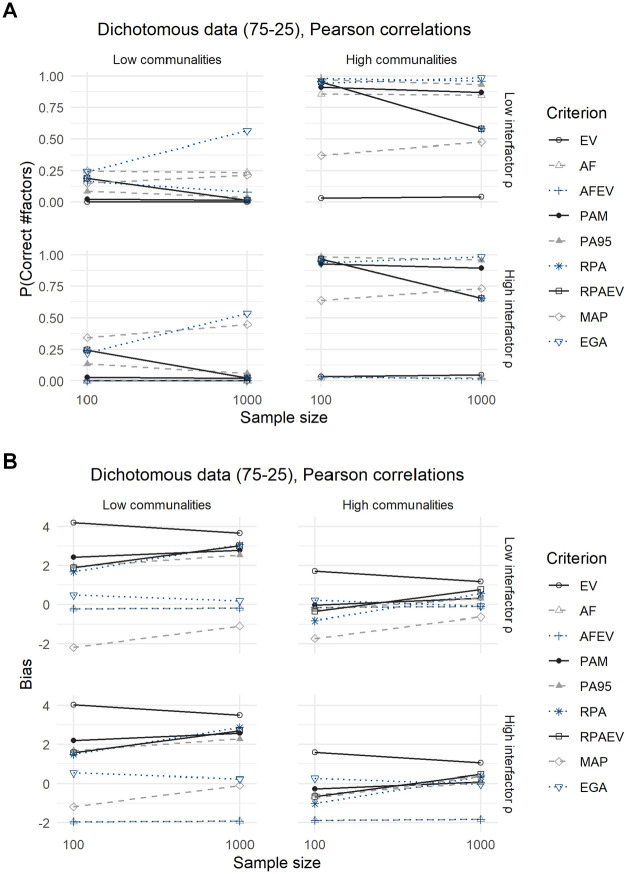
Results for Dichotomous Data With a 75–25 Split, Using Pearson Correlations: (A) Expected Probability of Correctly Predicting the Number of Factors and (B) Expected Bias.

**Figure 6.**
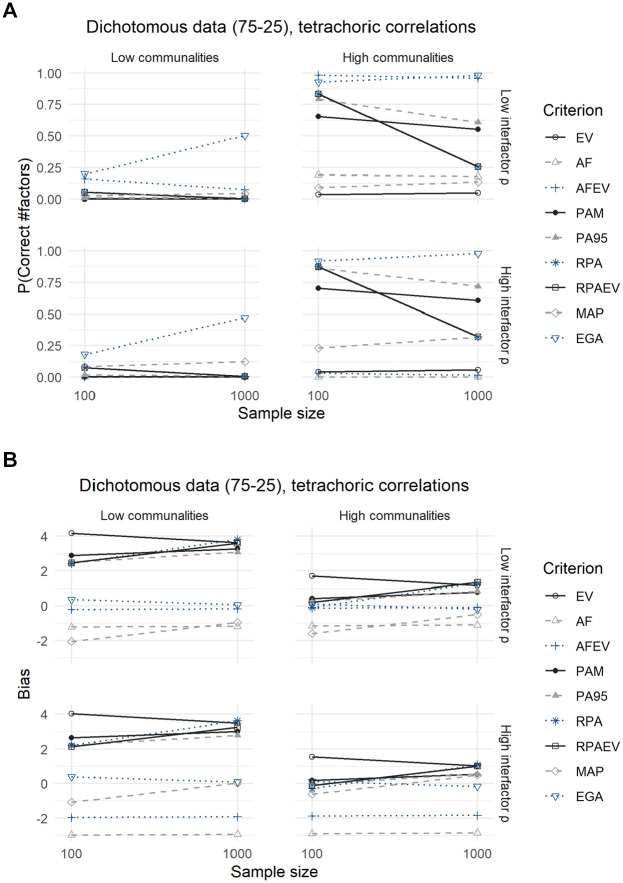
Results for Dichotomous Data With a 75–25 Split, Using Tetrachoric Correlations: (A) Expected Probability of Correctly Predicting the Number of Factors and (B) Expected Bias.

Compared with the analyses of the dichotomous data with a 50–50 split, conclusions are identical for the dichotomous data with a 75–25 split between zeroes and ones, as displayed in Figures 5 and 6 and their corresponding Tables 7 and 8.

**Table 7.** Results for Dichotomous Data With a 75–25 Split, Using Pearson Correlations.

A. Expected probability of correctly predicting the number of factors											
Communalities	Interfactor correlation	*N*	EV	AF	AFEV	PAM	PA95	RPA	RPAEV	MAP	EGA
Low	Low	100	0	0.25	0.16	0.02	0.09	0.19	0.19	0.15	0.24
Low	Low	1,000	0	0.23	0.08	0.01	0.04	0.02	0.02	0.21	0.57
Low	High	100	0	0	0	0.03	0.13	0.24	0.24	0.34	0.22
Low	High	1,000	0	0	0	0.02	0.06	0.02	0.02	0.45	0.54
High	Low	100	0.03	0.86	0.98	0.91	0.97	0.95	0.95	0.37	0.94
High	Low	1,000	0.04	0.85	0.96	0.87	0.93	0.58	0.58	0.48	0.99
High	High	100	0.03	0.02	0.03	0.93	0.98	0.97	0.97	0.64	0.94
High	High	1,000	0.05	0.02	0.01	0.89	0.96	0.65	0.65	0.73	0.98
B. Expected bias											
Communalities	Interfactor correlation	*N*	EV	AF	AFEV	PAM	PA95	RPA	RPAEV	MAP	EGA
Low	Low	100	4.18	−0.23	−0.23	2.44	1.96	1.68	1.89	−2.19	0.48
Low	Low	1,000	3.65	−0.19	−0.19	2.8	2.55	3.07	3.02	−1.09	0.19
Low	High	100	4.04	−1.97	−1.97	2.2	1.68	1.48	1.57	−1.19	0.52
Low	High	1,000	3.51	−1.93	−1.93	2.56	2.28	2.87	2.7	−0.09	0.23
High	Low	100	1.72	−0.15	−0.15	−0.04	−0.28	−0.83	−0.35	−1.73	0.22
High	Low	1,000	1.19	−0.11	−0.11	0.32	0.32	0.56	0.78	−0.63	−0.08
High	High	100	1.58	−1.89	−1.89	−0.27	−0.55	−1.03	−0.67	−0.73	0.26
High	High	1,000	1.05	−1.85	−1.85	0.09	0.04	0.36	0.45	0.37	−0.04

*Note.* EV = Kaiser criterion; AF = acceleration factor alternative to the scree plot; AFEV = acceleration factor combined with the Kaiser criterion; PAM = parallel analysis based on the mean eigenvalues; PA95 = parallel analysis based on the 95th percentile of eigenvalues; RPA = revised parallel analysis based on the 95th percentile of eigenvalues; RPAEV = revised parallel analysis based on the 95th percentile of eigenvalues combined with the Kaiser criterion; MAP = minimum average partial method; EGA = exploratory graph analysis.

**Table 8.** Results for Dichotomous Data With a 75–25 Split, Using Pearson Correlations.

A. Expected probability of correctly predicting the number of factors											
Communalities	Interfactor correlation	*N*	EV	AF	AFEV	PAM	PA95	RPA	RPAEV	MAP	EGA
Low	Low	100	0	0.01	0.16	0	0.01	0.06	0.06	0.03	0.2
Low	Low	1,000	0	0.01	0.08	0	0	0	0	0.04	0.5
Low	High	100	0	0	0	0.01	0.02	0.07	0.07	0.08	0.18
Low	High	1,000	0	0	0	0	0.01	0.01	0.01	0.12	0.47
High	Low	100	0.04	0.19	0.98	0.65	0.79	0.83	0.83	0.09	0.93
High	Low	1,000	0.05	0.18	0.96	0.55	0.61	0.25	0.25	0.14	0.98
High	High	100	0.04	0	0.03	0.7	0.86	0.87	0.87	0.23	0.92
High	High	1,000	0.06	0	0.01	0.61	0.72	0.32	0.32	0.32	0.98
B. Expected bias											
Communalities	Interfactor correlation	*N*	EV	AF	AFEV	PAM	PA95	RPA	RPAEV	MAP	EGA
Low	Low	100	4.15	−1.23	−0.21	2.89	2.48	2.42	2.45	−2.06	0.35
Low	Low	1,000	3.62	−1.19	−0.17	3.25	3.07	3.81	3.58	−0.96	0.06
Low	High	100	4.01	−2.97	−1.96	2.66	2.2	2.21	2.13	−1.06	0.39
Low	High	1,000	3.48	−2.93	−1.91	3.02	2.8	3.6	3.25	0.04	0.1
High	Low	100	1.69	−1.15	−0.14	0.42	0.24	−0.09	0.21	−1.6	0.09
High	Low	1,000	1.16	−1.11	−0.09	0.78	0.84	1.3	1.33	−0.5	−0.21
High	High	100	1.55	−2.89	−1.88	0.18	−0.03	−0.3	−0.11	−0.61	0.13
High	High	1,000	1.02	−2.85	−1.84	0.54	0.56	1.1	1.01	0.49	−0.17

*Note.* EV = Kaiser criterion; AF = acceleration factor alternative to the scree plot; AFEV = acceleration factor combined with the Kaiser criterion; PAM = parallel analysis based on the mean eigenvalues; PA95 = parallel analysis based on the 95th percentile of eigenvalues; RPA = revised parallel analysis based on the 95th percentile of eigenvalues; RPAEV = revised parallel analysis based on the 95th percentile of eigenvalues combined with the Kaiser criterion; MAP = minimum average partial method; EGA = exploratory graph analysis.

The MAP criterion performs significantly better when using Pearson correlations in terms of both the expected probability of predicting the correct number of factors to retain as well as the expected bias, but loses this advantage when employing tetrachoric correlations. In addition, using tetrachoric correlations leads to a decrease in the expected accuracies of all criteria, but relative performances remain the same. The underperformance of the MAP criterion is again due to its tendency to underextract in this case.

## Discussion

While many criteria have been developed to aid the choice of the number of factors to retain, we show that the most popular ones, such as the Kaiser criterion and scree plot, underperform. Studies by both Yang and Xia (2015) and Ruscio and Roche (2012) come to the same conclusions regarding the performance of the Kaiser criterion. We do, however, notice a better performance of the scree plot in the presence of high communalities from major factors and low interfactor correlations.

Both parallel analysis based on the mean eigenvalue and on the 95th percentile perform well in situations with high communalities from major factors. Research by Auerswald and Moshagen (2019), Cho et al. (2009), Crawford et al. (2010), Golino, Shi, et al. (2020) and Ruscio and Roche (2012) also support these conclusions. Yet, none of these studies point to the severe underperformance of parallel analysis when variables are only weakly influenced by their common factors. Only in small samples can revised parallel analysis (Green et al., 2012) alleviate some of the concerns regarding the accuracy of traditional parallel analysis. The study by Green et al. (2016) that indicates the preference of this former method in almost all circumstances can therefore not be endorsed. Auerswald and Moshagen (2019) already found this result for revised parallel analysis based on the reduced correlation matrix, and our results confirm it for the variant based on the full correlation matrix. In addition, revised parallel analysis shows accuracies that decrease as sample size increases. In general, however, larger sample sizes increase the accuracy of the criteria considered, which is in line with results obtained by Li et al. (2020).

EGA performs at least equally well as the criteria considered, yet usually outperforms them. While Golino, Shi, et al. (2020) also find that this criterion can match the performance of traditional parallel analysis, our results favor the former even more: Even in circumstances where most other criteria fail, is EGA able to achieve reasonable accuracy with larger sample sizes.^1^ Only in the case of binary data and analysis with Pearson correlations, can the minimum average partial method outperform EGA when faced with small samples and low communalities from major factors.

While tetrachoric correlations are usually recommended because they produce unbiased estimates of the relationships among the latent underlying continuous variables (Kirk, 1973; Olsson, 1979), we find that the use of tetrachoric correlations worsens the performance of all criteria when applied to binary data. Yet, the relative performances remain identical to the continuous case. Determining the number of factors to retain is therefore preferably done using the Pearson correlation matrix. It is important to note that the preference for Pearson correlations only applies to the determination of the optimal number of factors to retain, not to the rest of the factor analytic process. Studies (e.g., Barendse et al., 2015; Kaltenhauser & Lee, 1976; Lee et al., 2012) have shown that when extracting loadings, polychoric correlations are still preferred when working with ordinal data. In case of binary data, tetrachoric correlations are therefore still recommended when estimating loadings. In addition, this result was not previously discussed by other factor retention simulation studies, given that most of these studies apply tetrachoric correlations directly and do not compare it with the performances under Pearson correlations (see Table 1). Only Cho et al. (2009) find a similar conclusion when studying the accuracy of parallel analysis and reason that this results from the fact that tetrachoric correlations tend to be higher. This causes the eigenvalues of these correlation matrices to be higher for the first components and smaller for the subsequent ones. Finally, we find that the split between zeroes and ones does not affect the accuracy of the factor retention criteria for binary data.

## Conclusion and Directions for Future Research

The simulation results give way to several recommendations for researchers (cf. Table 9). First, when working with continuous data, EGA would seem the preferred criterion in all circumstances. In addition, researchers less familiar with this technique or its implementation might rely on revised parallel analysis (with or without the added use of the Kaiser criterion) in small samples. If the researcher is confident about the construction of his or her factors in that the manifest variables are all highly related to their accompanying latent variable in addition to disposing of a large sample, traditional parallel analysis (either based on mean or the 95th percentile of the eigenvalues) is also a good option.^2^

**Table 9.** Overview of Results: Recommended Procedure to Use in Each of the Scenarios.

	Low interfactor correlation	High interfactor correlation
Low communalities	- EGA	
	- RPA/RPAEV if small samples	
	- MAP if small samples and Pearson correlations	
High communalities	- AF/AFEV/EGA/PAM/PA95	- EGA/PAM/PA95
	- RPA/RPAEV if small samples	- RPA/RPAEV if small samples

*Note.* EGA = exploratory graph analysis; RPA = revised parallel analysis based on the 95th percentile of eigenvalues; RPAEV = revised parallel analysis based on the 95th percentile of eigenvalues Kaiser criterion; MAP = minimum average partial method; AF = acceleration factor alternative to the scree plot; AFEV = acceleration factor combined with the Kaiser criterion; PAM = parallel analysis based on the mean eigenvalues; PA95 = parallel analysis based on the 95th percentile of eigenvalues; RPAEV = revised parallel analysis based on the 95th percentile of eigenvalues combined with the Kaiser criterion.

Dichotomous data, either with a 50–50 or 75–25 split between zeroes and ones, can additionally benefit from the use of the minimum average partial method if analyzed with Pearson correlations. The MAP can be a valid, but less effective, alternative to the EGA method in case the researcher is more familiar with the former. Yet, this criterion loses its edge when analyzing dichotomous data with tetrachoric correlations. In addition, the use of this kind of correlation lowers the performance of all criteria.

But even when adhering to these guidelines, the number of factors extracted might not be correct. Underextraction compresses variables into a smaller factor space which leads to loss of information, neglect of important factors, distorted results, and increased error in the loadings. On the contrary, overextraction diffuses variables across a larger space, resulting in splitting of factors (Auerswald & Moshagen, 2019; Hayton et al., 2004). Underextraction is therefore a bigger problem and must be avoided at all costs. When multiple criteria give different answers, it is hence safer to choose the largest estimate of the optimal number of factors.

Overall, these results unveil opportunities for applied researchers: While Dolnicar et al. (2011) show that a binary response format saves respondent time and is perceived simpler while not influencing reliability or interpretations of results, our results show that at least part of the EFA process can be executed without loss of accuracy.

Yet, the focus on binary data has the inherent limitation to be only of moderate use in practice as Likert-type scales are still more often employed. Whether such data are suited for analysis using traditional factor retention criteria is still debated (Holgado-Tello et al., 2010) and therefore worth further investigation.

The data simulation procedure employed (Hong, 1999; Tucker et al., 1969) is considered standard in EFA research, yet also has its limitations. Most noticeably the fact that the sampled data follow a multivariate normal distribution. Auerswald and Moshagen (2019), however, show this does not influence the accuracy of these criteria in case of continuous data.

In addition, we have kept the degree of overdetermination constant, yet previous research has pointed to interplay between this degree and the sample size (e.g., Gaskin & Happell, 2014). Conclusions drawn regarding the latter should therefore be viewed in the context of our study. Repeating the same study, but varying the number of factors and variables per factor, is therefore another possibly fruitful avenue for future research. Other fixed^3^ characteristics of the simulation design, such as the number of minor factors, common ratio, and correlations between major and minor factors, were all kept constant, following other authors. Yet, future research would benefit from also assessing the impact of these variables on the performance of factor analysis.

The criteria and methods studied were largely based on an overview of current academic research, yet many more exist. Further research would profit from considering more, maybe less well-known procedures.

A last recommendation is the execution of a simulation study that takes into account more complex factor structures, given that this study only examines data with a loading matrix that satisfies perfect simple structure. Auerswald and Moshagen (2019) argue that cross-loadings should have a beneficial effect on both accuracy and bias because they increase the explained variance, yet Li et al. (2020) find the opposite. These last authors regard cross-loadings as a form of modeling error, as these are loadings on “minor factors.” We have also included “minor factors,” yet not by modeling cross-loadings on major factors. This could explain why the accuracies we obtain are lower than those found by Auerswald and Moshagen (2019). Yet, these scenarios remain a recommendation for future research.

## Supplemental Material

sj-pdf-1-epm-10.1177_00131644211059089 – Supplemental material for Exploratory Graph Analysis for Factor Retention: Simulation Results for Continuous and Binary Data Click here for additional data file. Supplemental material, sj-pdf-1-epm-10.1177_00131644211059089 for Exploratory Graph Analysis for Factor Retention: Simulation Results for Continuous and Binary Data by Tim Cosemans, Yves Rosseel and Sarah Gelper in Educational and Psychological Measurement

## Notes

1. This discrepancy might be explained by the fact that Golino, Shi, et al. (2020) do not allow for the “lack-of-fit” commonly found when fitting the factor model to real-world data (Hong, 1999; MacCallum & Tucker, 1991; Tucker et al., 1969).

2. These analyses can even be conducted using an online application like https://analytics.gonzaga.edu/parallelengine/.

3. Although discrete, we do believe the variable input parameters considered in the simulation design cover the most realistic scenarios.
