# Supplementary material for: Exploratory Graph Analysis for Factor Retention: Simulation Results for Continuous and Binary Data
Source: Educ Psychol Meas. 2021 Dec 28;82(5):880–910. doi: 10.1177/00131644211059089 (PMC9386885; doi:10.1177/00131644211059089)
Supplement: sj-pdf-1-epm-10.1177_00131644211059089 – Supplemental material for Exploratory Graph Analysis for Factor Retention: Simulation Results for Continuous and Binary Data [file sj-pdf-1-epm-10.1177_00131644211059089.pdf]

Exploratory graph analysis for factor retention: Simulation results for continuous and binary data - supplementary materials

Wed Dec 29 16:24:57 2021

In the supplementary materials, we detail the data simulation procedure discussed in the main manuscript. In addition, we present the matrices that result from the four scenarios we built and the full regression results that follow from our analyses.

Appendix

1 Data simulation procedure

1.1 Formal and simulation model

In order to compare various factor retention criteria, extraction methods and rotation procedures, the real underlying structure of the data at hand should be known. In this paragraph, we describe the procedure that generates population correlation matrices from which the data for the subsequent factor analysis were sampled. This procedure relies on the simulation model, proposed by tucker1969. This model assumes the existence of a major domain, that contains the factors of influence the researcher wishes to study, unique factors, that account for variable specific influences and measurement errors and (infinitely) many minor factors not modelled by the researcher. More specifically, there are three types of factors, denoted by m_s (m_1 for major, m_2 for minor and m_3 for unique factors). The number of each of these factors is denoted by M_s. If we assume the presence of J measured variables, tucker1969 propose there exists a matrix $\mathbf{A_s}$ (of order $J \times M_s$) for each type of factor with actual input factor loadings and a matrix $\mathbf{A_s^*}$ that is formed by adjusting the rows of $\mathbf{A_s}$ to unit length vectors such that

$$\mathbf{P_s} = \mathbf{A_s^* A_s^{*\prime}} \tag{1}$$

This matrix $\mathbf{P_s}$ is a square matrix of order J with $diag(\mathbf{P}) = \mathbf{I}$. The simulated correlation matrix can then be written as

$$\mathbf{R_{YY}} = \mathbf{B_1 P_1 B_1} + \mathbf{B_2 P_2 B_2} + \mathbf{B_3 P_3 B_3} \tag{2}$$

where $\mathbf{B_s}$ is a diagonal matrix with entries b_{sj}. These entries are restricted such that

$$b_{1j}^2 + b_{2j}^2 + b_{3j}^2 = 1 \tag{3}$$

so that the simulated population correlation matrix $\mathbf{R_{YY}}$ contains only unities on its diagonal. The matrix $\mathbf{A_s}$ and $\mathbf{R_{YY}}$ can then be defined as

$$\mathbf{A_s} = \mathbf{B_s A_s^*} \tag{4}$$

$$\mathbf{R_{YY}} = \mathbf{A_1 A_1'} + \mathbf{A_2 A_2'} + \mathbf{A_3 A_3'} \tag{5}$$

The coefficients in the matrix $\mathbf{B_s}$ are important parameters: they regulate the proportion of the variances of the variables derived from the three types of factors. $(\mathbf{B_1})^2$, for example, contains the communalities of the variables from major factors. And if we set $\mathbf{B_2} = \mathbf{0}$, the simulation model equals the formal model discussed above [Tucker, Koopman LinnTucker.1969]. Comparing the fundamental equation from the main manuscript (Equation 3) and Equation 5, we can see that the matrix $\mathbf{A_1}$ is equal to the matrix L. In addition, the matrix resulting from the product $\mathbf{A_3 A_3'}$ equals the previously discussed matrix Y.

The procedure by tucker1969 does not allow for correlation between major factors however, yet in the social sciences this is often the case. hong1999 therefore introduces $\mathbf{L} = [\mathbf{A_1} \;\; \mathbf{A_2}]$, the super loading matrix, and $\mathbf{C}$, the matrix of factor correlations. The latter of which can be partitioned as follows

$$\mathbf{C} = \begin{bmatrix} \mathrm{Q} & ¡ \\ ¡' & \mathrm{G} \\ & \end{bmatrix} \tag{6}$$

where Q is an $M_1 \times M_1$ matrix of correlations among major factors (previously denoted by $\mathbf{R_{HH}}$), G is an $M_2 \times M_2$ matrix of correlations among minor factors and ¡ is an $M_1 \times M_2$ matrix of correlations among major and minor factors. The population correlation matrix can then be written as

$$\mathbf{R} = \mathbf{LCL'} + \mathbf{A_3 A_3'} = \mathbf{LCL'} + \mathrm{Y} \tag{7}$$

Given this population correlation matrix, we can use the procedure by kaiser1962 to simulate data with sample size N as described in the main body of the paper.

1.2 Major factor loadings

In accordance with tucker1969, the generation of the actual input factor loadings starts with the generation of the matrix $\mathbf{A_1}$ containing the conceptual input factor loadings. These reflect the ideas of the researcher regarding the importance of the variables to the different factors. tucker1969 proceed by randomly allocating an integer between zero and two to each of the cells, restricting the sum of each row to equal two. In this study, we require the actual input factor loadings ($\mathbf{A_1}$) to have perfect simple structure, following the applications of this method in

literature [Briggs MacCallumBriggs MacCallum2003, De~Winter DodouDe~Winter Dodou2012, De~Winter DodouDe~Winter Dodou2016, MacCallum, Widaman, Zhang HongMacCallum.1999, Pearson MundformPearson Mundform2010]. We therefore set only one cell for each variable different from zero (at two, thereby still fulfilling the restriction set by tucker1969; see Table 1). These loadings are then multiplied by the inverse of the norm of each row $\left(\left(\sum_{m_1} a_{jm_1}^2\right)^{-1/2}\right)$, so that the resulting rows of $\mathbf{A}_1^*$ each have unit length. In the final step, $\mathbf{A}_1^*$ is premultiplied by $\mathbf{B}_1$ to yield $\mathbf{A}_1$ or `L`, the actual input factor loadings[1].

Table 1: Conceptual input factor loadings

	FACTOR 1	FACTOR 2	FACTOR 3
VAR 1	2	0	0
VAR 2	2	0	0
VAR 3	2	0	0
VAR 4	2	0	0
VAR 5	2	0	0
VAR 6	2	0	0
VAR 7	0	2	0
VAR 8	0	2	0
VAR 9	0	2	0
VAR 10	0	2	0
VAR 11	0	2	0
VAR 12	0	2	0
VAR 13	0	2	0
VAR 14	0	0	2
VAR 15	0	0	2
VAR 16	0	0	2
VAR 17	0	0	2
VAR 18	0	0	2
VAR 19	0	0	2

[1] $\mathbf{B}_1$ is a diagonal matrix with elements equal to the positive square roots of the chosen communalities from major factors.

VAR 20	0	0	2

1.3 Minor factor loadings

A matrix with loadings on several other, unmodelled, factors is generated by drawing independent random standard normal deviates ($\mu = 0$, $\sigma = 1$). These entries are multiplied columnwise by a constant $(1-\varepsilon)^{(m_2-1)}$ to form a decreasing geometric series. Given that this constant decreases in size as we progress throughout the columns of the matrix, so do the loadings. The infinite number of minor factors can therefore be approximated by a finite matrix. In addition, the rows of the resulting matrix are normalised to have unit length [HongHong1999, MacCallum TuckerMacCallum Tucker1991, Tucker, Koopman LinnTucker.1969]. More specifically, if $\mathbf{A}_2$ contains the random standard normal deviates and a_{jm_2} is the element of $\mathbf{A}_2$ in row j and column m_2

$$a^{**}_{jm_2} = a_{jm_2}(1-\varepsilon)^{(m_2-1)} \tag{8}$$

$$a^{*}_{jm_2} = a^{**}_{jm_2}\left(\sum_{m_2} a^{**2}_{jm_2}\right)^{-1/2} \tag{9}$$

Then left to decide is the size of the elements in the diagonal matrix $\mathbf{B}_2$. Following tucker1969 we set each element $b_{2j} = \sqrt{\left(1-b^2_{1j}\right)/2}$. In a final step we, again, premultiply $\mathbf{A}^*_2$ by $\mathbf{B}_2$ to yield $\mathbf{A}_2$, the factor loadings on minor factors.

1.4 Unique factor loadings

Given that the Tucker-Koopman-Linn procedure tucker1969 assumes uncorrelated factors, their computation of the unique factor loadings is not applicable in this study. Following hong1999, we compute the unique variance matrix Y as

$$Y = \mathbf{I} - diag\left(\mathbf{LCL}'\right) \tag{10}$$

using the restriction from Equation 3, where $\mathbf{I}$ is an identity matrix.

These equations allow for the construction of the population correlation matrix. Further details on the sampling procedure and the construction of the scenarios are given in the main body of the paper.

2 Correlation and factor loading matrices

Table 2: High communalities from major factors, high interfactor correlations - Actual input factor loadings

	FACTOR 1	FACTOR 2	FACTOR 3
VAR 1	0.89	0	0
VAR 2	0.77	0	0
VAR 3	0.77	0	0
VAR 4	0.77	0	0
VAR 5	0.77	0	0
VAR 6	0.84	0	0
VAR 7	0	0.77	0
VAR 8	0	0.77	0
VAR 9	0	0.84	0
VAR 10	0	0.84	0
VAR 11	0	0.84	0
VAR 12	0	0.77	0
VAR 13	0	0.89	0
VAR 14	0	0	0.77
VAR 15	0	0	0.89
VAR 16	0	0	0.84
VAR 17	0	0	0.84
VAR 18	0	0	0.84
VAR 19	0	0	0.84
VAR 20	0	0	0.89

Table 3: High communalities from major factors, high interfactor correlations - Population correlation matrix

!

	VAR 1	VAR 2	VAR 3	VAR 4	VAR 5	VAR 6	7
VAR 1	1	0.71	0.77	0.62	0.68	0.78	
VAR 2	0.71	1	0.54	0.52	0.68	0.64	
VAR 3	0.77	0.54	1	0.50	0.54	0.71	
VAR 4	0.62	0.52	0.50	1	0.55	0.57	
VAR 5	0.68	0.68	0.54	0.55	1	0.60	
VAR 6	0.78	0.64	0.71	0.57	0.60	1	
VAR 7	0.22	0.32	0.22	0.35	0.35	0.27	
VAR 8	0.32	0.21	0.26	0.46	0.19	0.27	
VAR 9	0.33	0.26	0.26	0.46	0.34	0.30	
VAR 10	0.46	0.31	0.41	0.29	0.25	0.39	
VAR 11	0.38	0.42	0.25	0.36	0.39	0.26	
VAR 12	0.28	0.31	0.24	0.33	0.43	0.29	
VAR 13	0.45	0.35	0.44	0.25	0.31	0.45	
VAR 14	0.38	0.25	0.41	0.17	0.30	0.43	
VAR 15	0.33	0.29	0.33	0.45	0.34	0.32	
VAR 16	0.37	0.24	0.41	0.37	0.24	0.30	
VAR 17	0.33	0.30	0.24	0.39	0.29	0.35	
VAR 18	0.35	0.34	0.21	0.41	0.40	0.30	
VAR 19	0.39	0.40	0.27	0.34	0.37	0.29	
VAR 20	0.38	0.44	0.26	0.35	0.42	0.32	

Table 4: High communalities from major factors, high interfactor correlations - Population

correlation matrix (continued)

!

	VAR 11	VAR 12	VAR 13	VAR 14	VAR 15	VAR 16	17
VAR 1	0.38	0.28	0.45	0.38	0.33	0.37	
VAR 2	0.42	0.31	0.35	0.25	0.29	0.24	
VAR 3	0.25	0.24	0.44	0.41	0.33	0.41	
VAR 4	0.36	0.33	0.25	0.17	0.45	0.37	
VAR 5	0.39	0.43	0.31	0.30	0.34	0.24	
VAR 6	0.26	0.29	0.45	0.43	0.32	0.30	
VAR 7	0.67	0.71	0.66	0.25	0.43	0.34	
VAR 8	0.65	0.58	0.66	0.18	0.43	0.40	
VAR 9	0.73	0.73	0.70	0.23	0.46	0.37	
VAR 10	0.71	0.55	0.83	0.31	0.34	0.41	
VAR 11	1	0.67	0.71	0.19	0.39	0.34	
VAR 12	0.67	1	0.65	0.28	0.40	0.28	
VAR 13	0.71	0.65	1	0.40	0.37	0.39	
VAR 14	0.19	0.28	0.40	1	0.62	0.62	
VAR 15	0.39	0.40	0.37	0.62	1	0.81	
VAR 16	0.34	0.28	0.39	0.62	0.81	1	
VAR 17	0.32	0.31	0.30	0.67	0.73	0.65	
VAR 18	0.43	0.41	0.30	0.56	0.77	0.63	
VAR 19	0.48	0.30	0.35	0.54	0.75	0.71	
VAR 20	0.47	0.38	0.36	0.62	0.79	0.71	

Table 5: High communalities from major factors, low interfactor correlations - Actual input

factor loadings

	FACTOR 1	FACTOR 2	FACTOR 3
VAR 1	0.89	0	0
VAR 2	0.77	0	0
VAR 3	0.77	0	0
VAR 4	0.77	0	0
VAR 5	0.77	0	0
VAR 6	0.84	0	0
VAR 7	0	0.77	0
VAR 8	0	0.77	0
VAR 9	0	0.84	0
VAR 10	0	0.84	0
VAR 11	0	0.84	0
VAR 12	0	0.77	0
VAR 13	0	0.89	0
VAR 14	0	0	0.77
VAR 15	0	0	0.89
VAR 16	0	0	0.84
VAR 17	0	0	0.84
VAR 18	0	0	0.84
VAR 19	0	0	0.84
VAR 20	0	0	0.89

Table 6: High communalities from major factors, low interfactor correlations - Population correlation matrix

!

	VAR 1	VAR 2	VAR 3	VAR 4	VAR 5	VAR 6	7
VAR 1	1	0.71	0.77	0.62	0.68	0.78	
- 0.02							
–0							
- 0.09							
- 0.01							
VAR 3	0.77	0.54	1	0.50	0.54	0.71	
- 0.04							
–0.070							
- 0.04							
VAR 5	0.68	0.68	0.54	0.55	1	0.60	
- 0.08							
VAR 6	0.78	0.64	0.71	0.57	0.60	1	
- 0.05							
- 0.13	0.02						
- 0.05	1	0.59	0.68	0.55			
VAR 8							
- 0.09							
- 0.11							
- 0.04							
- 0.07	0.13	0.02					
- 0.01	0.09						
- 0.08	0.04	0.55	0.69	0.69	1		
VAR 11	0.01	0.10					

- 0.09	0.67	0.65	0.73	0.71
VAR 12				
- 0.06	0.03	0.13		
	–0.			
- 0.09				
	-			
- 0.05	0.11			
- 0.002	0.11			
- 0.12				
- 0.01				
VAR 15				
- 0.06				
- 0.01				
- 0.03				
VAR 16				
- 0.09	0.09	0.04		
- 0.05	0.01	0.07	0.02	0.06
VAR 17				
- 0.03				
- 0.03				
- 0.01				
- 0.02	0.02			
- 0.05	0.01	0.03	0.09	
- 0.06	0.02	0.05		

- 0.01	0.02	0.03	
VAR 20			
- 0.09	0.004	0.07	
- 0.05			
- 0.04			

Table 7: High communalities from major factors, low interfactor correlations - Population correlation matrix (continued)

!

	VAR 11	VAR 12	VAR 13	VAR 14	VAR 15	VAR 16	17
VAR 1	0.01						
- 0.07							
- 0.04							
- 0.02							
VAR 2	0.10	0.01	0.004				
- 0.06							
- 0.03	0.02	0.08	0.10				
VAR 3							
- 0.06	0.09	0.11					
- 0.08							
- 0.06							
- 0.09							
–0.1[illegible]							

- 0.03						
- 0.01						
- 0.03	0.08	0.05	0.07			
VAR 6						
- 0.04	0.07	0.11				
- 0.05						
- 0.05						
- 0.05						
VAR 7	0.67	0.71	0.66			
	–0.050					
- 0.12	0.08	0.07	0.01	0.03		
- 0.05						
VAR 9	0.73	0.73	0.70			
- 0.01	0.09	0.02				
- 0.01						
- 0.09						
- 0.04						
VAR 11	1	0.67	0.71			
- 0.01						
- 0.02	0.05					
- 0.01	0.09					
- 0.03	0.02					
- 0.08						
- 0.04						
VAR 14						

- 0.02	0.06	1	0.62	0.62	0.67	0.56	0.
VAR 15	0.01	0.05					
–							
- 0.01							
–0.							
- 0.03							
- 0.08	0.67	0.73	0.65	1	0.74	0.68	0.
VAR 18	0.08	0.09					
–0.							
- 0.02							
–0.03							
- 0.04	0.62	0.79	0.71	0.76	0.81	0.83	1

Table 8: Low communalities from major factors, high interfactor correlations - Actual input factor loadings

	FACTOR 1	FACTOR 2	FACTOR 3
VAR 1	0.45	0	0
VAR 2	0.63	0	0
VAR 3	0.45	0	0
VAR 4	0.55	0	0
VAR 5	0.45	0	0
VAR 6	0.63	0	0
VAR 7	0	0.63	0
VAR 8	0	0.55	0
VAR 9	0	0.55	0
VAR 10	0	0.63	0
VAR 11	0	0.63	0
VAR 12	0	0.45	0

VAR 13	0	0.45	0
VAR 14	0	0	0.45
VAR 15	0	0	0.55
VAR 16	0	0	0.55
VAR 17	0	0	0.55
VAR 18	0	0	0.55
VAR 19	0	0	0.63
VAR 20	0	0	0.45

Table 9: Low communalities from major factors, high interfactor correlations - Population correlation matrix

!

	VAR 1	VAR 2	VAR 3	VAR 4	VAR 5	VAR 6	VAR 7
VAR 1	1	0.33	0.41	0.06	0.16	0.37	
- 0.01	0.39						
VAR 2	0.33	1	0.18	0.21	0.43	0.38	
VAR 3	0.41	0.18	1	0.05	0.08	0.41	
–0.020.32*VAR*40.060.2							
- 0.08	0.16						
–0.01*VAR*							
- 0.17	0.23	0.003	0.25	0.22	0.11	1	0.
VAR 8	0.06	0.02	0.06	0.43			
- 0.01	0.04						
–0.020.42							
- 0.01	0.28	0.23	0.43	0.33	1		
VAR 11	0.16	0.37					

–0.							
- 0.10	0.15						
–0.010.1							
- 0.12	0.01	0.35	0.20	0.16	0.10	0.52	
VAR 14	0.19	0.05	0.31				
- 0.09							
- 0.13	0.04	0.07	0.40	0.10	0.02	0.37	0.
VAR 16	0.12	0.01	0.31	0.23			
- 0.003	0.13						
- 0.01							
VAR 18	0.06	0.21					
–0.130.330.300.070.19							
- 0.14	0.13	0.30					
- 0.001	0.10	0.02					

Table 10: Low communalities from major factors, high interfactor correlations - Population correlation matrix (continued)

!

	VAR 11	VAR 12	VAR 13	VAR 14	VAR 15	VAR 16	17
VAR 1	0.16						
- 0.13	0.12						
–0.0030.060.190.0							
- 0.001							
- 0.05							
- 0.14							
VAR 4	0.24	0.17					

- 0.13	0.40	0.23	0.27	0.33	0.21	0.13	
VAR 5	0.28	0.36	0.01	0.10	0.10		
–0.060.050.30							
- 0.01							
VAR 7	0.44	0.48	0.20	0.05	0.37	0.20	
VAR 8	0.35	0.21	0.16				
- 0.001							
VAR 9	0.42	0.42	0.10				
–0.090.390.2							
- 0.01	0.06	0.26	0.02				
VAR 11	1	0.33	0.18				
–0.130.210.160.110.36							
- 0.001	0.19						
- 0.12	0.07						
- 0.13	0.07	0.26	1	0.05	0.18	0.28	0.
VAR 15	0.21	0.27					
–0.0010.0510.490.260.							
- 0.11	0.28	0.26	0.18	1	0.40	0.31	0.
VAR 18	0.36	0.31					
–0.120.060.350.140.40							
- 0.05	0.004	0.22	0.12	0.29	0.44	0.51	1

Table 11: Low communalities from major factors, low interfactor correlations - Actual input factor loadings

	FACTOR 1	FACTOR 2	FACTOR 3
VAR 1	0.45	0	0
VAR 2	0.63	0	0
VAR 3	0.45	0	0
VAR 4	0.55	0	0

VAR 5	0.45	0	0
VAR 6	0.63	0	0
VAR 7	0	0.63	0
VAR 8	0	0.55	0
VAR 9	0	0.55	0
VAR 10	0	0.63	0
VAR 11	0	0.63	0
VAR 12	0	0.45	0
VAR 13	0	0.45	0
VAR 14	0	0	0.45
VAR 15	0	0	0.55
VAR 16	0	0	0.55
VAR 17	0	0	0.55
VAR 18	0	0	0.55
VAR 19	0	0	0.63
VAR 20	0	0	0.45

Table 12: Low communalities from major factors, low interfactor correlations - Population correlation matrix

!

	VAR 1	VAR 2	VAR 3	VAR 4	VAR 5	VAR 6	VAR 7
VAR 1	1	0.33	0.41	0.06	0.16	0.37	
- 0.06							
–0							
- 0.15							

- 0.02							
VAR 3	0.41	0.18	1	0.05	0.08	0.41	
- 0.07							
	–0.140						
- 0.07							
VAR 5	0.16	0.43	0.08	0.15	1	0.19	
- 0.15							
VAR 6	0.37	0.38	0.41	0.20	0.19	1	
- 0.10							
- 0.31	0.03						
- 0.09	1	0.34	0.41	0.23			
VAR 8							
- 0.15							
- 0.21							
- 0.13							
- 0.14	0.27	0.03					
- 0.02	0.18						
- 0.15	0.08	0.23	0.43	0.33	1		
VAR 11	0.02	0.17					
- 0.17	0.44	0.35	0.42	0.42			
VAR 12							
- 0.11	0.05	0.26					
	–0						
- 0.24							
	-						
- 0.09	0.21						
- 0.004	0.22						

-0.22				
-0.03				
VAR 15				
-0.13				
-0.02				
-0.08				
VAR 16				
-0.17	0.19	0.08		
-0.10	0.02	0.15	0.05	0.13
VAR 17				
-0.05				
-0.07				
-0.02				
-0.06	0.04			
-0.10	0.02	0.06	0.21	
-0.12	0.04	0.10		
-0.02	0.05	0.06		
VAR 20				
-0.24	0.01	0.20		
-0.12				
-0.12				

Table 13: Low communalities from major factors, low interfactor correlations - Population correlation matrix (continued)

!

	VAR 11	VAR 12	VAR 13	VAR 14	VAR 15	VAR 16	VAR 17
VAR 1	0.02						
- 0.26							
- 0.13							
- 0.08							
VAR 2	0.17	0.01	0.01				
- 0.13							
- 0.05	0.04	0.14	0.24				
VAR 3							
- 0.11	0.26	0.21					
- 0.17							
- 0.12							
- 0.24							
	–0.2						
- 0.09							
- 0.02							
- 0.07	0.17	0.10	0.20				
VAR 6							
- 0.08	0.21	0.22					
- 0.10							

-0.10							
-0.15							
VAR 7	0.44	0.48	0.20				
	–0.09[illegible]						
-0.22	0.20	0.15	0.02	0.06			
-0.12							
VAR 9	0.42	0.42	0.10				
-0.02	0.21	0.05					
-0.03							
-0.19							
-0.12							
VAR 11	1	0.33	0.18				
-0.01							
-0.03	0.15						
-0.02	0.19						
-0.12	0.06						
-0.24							
-0.15							
VAR 14							
-0.03	0.16	1	0.05	0.18	0.28	0.06	0.[illegible]
VAR 15	0.03	0.15					
	–[illegible]						
-0.01							
	–0.[illegible]						
-0.06							
-0.24	0.28	0.26	0.18	1	0.40	0.31	0.[illegible]
VAR	0.18	0.19					

18							
–0.							
- 0.05							
–0.07							
- 0.15	0.004	0.22	0.12	0.29	0.44	0.51	1

3 Full regression results

	Dependent variable:	
	Bias (OLS)	Percentage correct (logistic)
	(1)	(2)
Sample size 1000	–0.534 ***	0.342 ***
	(0.010)	(0.065)
High interfactor correlation	–0.138 ***	0.112 *
	(0.010)	(0.064)
High communalities	–2.460 ***	14.645
	(0.010)	(27.612)
Dichotomous data (50-50)	0.336 ***	–1.091 ***
	(0.015)	(0.091)

Dichotomous data (75-25)	0.489 ***	–0.982 ***
	(0.015)	(0.089)
Tetrachoric correlations	–0.027 **	0.200 **
	(0.011)	(0.081)
EGA	–3.236 ***	16.415
	(0.020)	(27.612)
MAP	–3.953 ***	13.734
	(0.020)	(27.612)
PA95	–1.565 ***	14.567
	(0.020)	(27.612)
PAM	–1.222 ***	13.676
	(0.020)	(27.612)
RPA	–1.485 ***	15.315
	(0.020)	(27.612)
RPAEV	–1.645 ***	15.315
	(0.020)	(27.612)
AF	–3.921 ***	15.920

	(0.020)	(27.612)
AFEV	–3.926***	15.316
	(0.020)	(27.612)
Sample size 1000*EGA	0.238***	1.063***
	(0.014)	(0.071)
Sample size 1000*MAP	1.634***	0.095
	(0.014)	(0.070)
Sample size 1000*PA95	1.130***	–1.247***
	(0.014)	(0.076)
Sample size 1000*PAM	0.895***	–0.768***
	(0.014)	(0.075)
Sample size 1000*RPA	1.925***	–3.037***
	(0.014)	(0.076)
Sample size 1000*RPAEV	1.659***	–3.037***
	(0.014)	(0.076)
Sample size 1000*AF	0.577***	–0.412***

	(0.014)	(0.076)
Sample size 1000*AFEV	0.577 ***	– 1.183 ***
	(0.014)	(0.085)
High interfactor correlation*EGA	0.177 ***	– 0.232 ***
	(0.014)	(0.070)
High interfactor correlation*MAP	1.132 ***	0.979 ***
	(0.014)	(0.070)
High interfactor correlation*PA95	– 0.136 ***	0.386 ***
	(0.014)	(0.075)
High interfactor correlation*PAM	– 0.097 ***	0.115
	(0.014)	(0.074)
High interfactor correlation*RPA	– 0.067 ***	0.207 ***
	(0.014)	(0.071)
High interfactor	– 0.186 ***	0.207 ***

correlation*RPAEV		
	(0.014)	(0.071)
High interfactor correlation*AF	–1.602 ***	–5.622 ***
	(0.014)	(0.111)
High interfactor correlation*AFEV	–1.604 ***	–7.568 ***
	(0.014)	(0.113)
High communalities*EGA	2.195 ***	–10.705
	(0.014)	(27.612)
High communalities*MAP	2.917 ***	–13.439
	(0.014)	(27.612)
High communalities*PA95	0.225 ***	–8.770
	(0.014)	(27.612)
High communalities*PAM	–0.013	–8.545
	(0.014)	(27.612)
High communalities*RPA	–0.048 ***	–10.186

	(0.014)	(27.612)
High communalities *RPAEV	0.217 ***	– 10.186
	(0.014)	(27.612)
High communalities *AF	2.538 ***	– 11.747
	(0.014)	(27.612)
High communalities *AFEV	2.537 ***	– 9.008
	(0.014)	(27.612)
Dichotomous data (50-50)*EGA	– 0.303 ***	0.808 ***
	(0.021)	(0.100)
Dichotomous data (75-25)*EGA	– 0.461 ***	0.549 ***
	(0.021)	(0.098)
Dichotomous data (50-50)*MAP	– 1.953 ***	2.990 ***
	(0.021)	(0.099)
Dichotomous data (75-25)*MAP	– 2.412 ***	2.634 ***
	(0.021)	(0.097)
Dichotomous data	–	1.740

(50-50)*PA95	0.583 ***	***
	(0.021)	(0.110)
Dichotomous data (75-25)*PA95	– 0.659 ***	1.172 ***
	(0.021)	(0.109)
Dichotomous data (50-50)*PAM	– 0.479 ***	1.288 ***
	(0.021)	(0.112)
Dichotomous data (75-25)*PAM	– 0.522 ***	0.655 ***
	(0.021)	(0.110)
Dichotomous data (50-50)*RPA	– 0.592 ***	1.614 ***
	(0.021)	(0.101)
Dichotomous data (75-25)*RPA	– 1.014 ***	1.341 ***
	(0.021)	(0.099)
Dichotomous data (50-50)*RPAEV	– 0.570 ***	1.614 ***
	(0.021)	(0.101)
Dichotomous data (75-25)*RPAEV	– 0.642 ***	1.341 ***

	(0.021)	(0.099)
Dichotomous data (50-50)*AF	–0.346 ***	1.156 ***
	(0.021)	(0.105)
Dichotomous data (75-25)*AF	–0.490 ***	1.069 ***
	(0.021)	(0.103)
Dichotomous data (50-50)*AFEV	–0.349 ***	1.254 ***
	(0.021)	(0.119)
Dichotomous data (75-25)*AFEV	–0.483 ***	1.159 ***
	(0.021)	(0.118)
Tetrachoric correlations*EGA	–0.101 ***	–0.460 ***
	(0.016)	(0.087)
Tetrachoric correlations*MAP	0.154 ***	–1.964 ***
	(0.016)	(0.086)
Tetrachoric correlations*PA95	0.547 ***	–2.369 ***

	(0.016)	(0.097)
Tetrachoric correlations*PAM	0.482 ***	–1.893 ***
	(0.016)	(0.092)
Tetrachoric correlations*RPA	0.763 ***	–1.593 ***
	(0.016)	(0.088)
Tetrachoric correlations*RPAEV	0.586 ***	–1.593 ***
	(0.016)	(0.088)
Tetrachoric correlations*AF	–0.973 ***	–3.420 ***
	(0.016)	(0.097)
Tetrachoric correlations*AFEV	0.043 ***	–0.228 **
	(0.016)	(0.099)
Intercept	3.691 ***	–17.121
	(0.014)	(27.612)
Observations	360,000	360,000

Note:	* $p < 0.1$; ** $p < 0.05$; *** $p < 0.01$

References

[Briggs MacCallumBriggs MacCallum2003] briggs2003Briggs, N. MacCallum, R. 2003. Recovery of weak common factors by maximum likelihood and ordinary least squares estimation Recovery of weak common factors by maximum likelihood and ordinary least squares estimation. Multivariate Behavioral Research38125–56.

[De~Winter DodouDe~Winter Dodou2012] dewinter2012De Winter, J C. Dodou, D. 2012. Factor recovery by principal axis factoring and maximum likelihood factor analysis as a function of factor pattern and sample size Factor recovery by principal axis factoring and maximum likelihood factor analysis as a function of factor pattern and sample size. Journal of Applied Statistics394695–710.

[De~Winter DodouDe~Winter Dodou2016] dewinter2016De Winter, J C. Dodou, D. 2016. Common factor analysis versus principal component analysis: a comparison of loadings by means of simulations Common factor analysis versus principal component analysis: a comparison of loadings by means of simulations. Communications in Statistics-Simulation and Computation451299–321.

[HongHong1999] hong1999Hong, S. 1999. Generating correlation matrices with model error for simulation studies in factor analysis: A combination of the Tucker-Koopman-Linn model and Wijsman's algorithm Generating correlation matrices with model error for simulation studies in factor analysis: A combination of the tucker-koopman-linn model and wijsman's algorithm. Behavior Research Methods, Instruments, & Computers314727–730.

[Kaiser DickmanKaiser Dickman1962] kaiser1962Kaiser, H. Dickman, K. 1962. Sample and population score matrices and sample correlation matrices from an arbitrary population correlation matrix Sample and population score matrices and sample correlation matrices from an arbitrary population correlation matrix. Psychometrika272179–182.

[MacCallum TuckerMacCallum Tucker1991] maccallum1991MacCallum, R. Tucker, L. 1991. Representing sources of error in the common-factor model: Implications for theory and practice. Representing sources of error in the common-factor model: Implications for theory and practice. Psychological Bulletin1093502–511.

[MacCallum, Widaman, Zhang HongMacCallum.1999] maccallum1999MacCallum, R., Widaman, K., Zhang, S. Hong, S. 1999. Sample size in factor analysis. Sample size in factor analysis. Psychological Methods4184–99.

[Pearson MundformPearson Mundform2010] pearson2010Pearson, R. Mundform, D. 2010. Recommended sample size for conducting exploratory factor analysis on dichotomous data Recommended sample size for conducting exploratory factor analysis on dichotomous data. Journal of Modern Applied Statistical Methods92359–368.

[Tucker, Koopman LinnTucker.1969] tucker1969Tucker, L., Koopman, R. Linn, R. 1969. Evaluation of factor analytic research procedures by means of simulated correlation matrices Evaluation of factor analytic research procedures by means of simulated correlation matrices. Psychometrika344421–459.
